# Artificial intelligence based assessment of treatment response in wet age related macular degeneration using paired OCT angiography

**DOI:** 10.1038/s41598-026-42999-7

**Published:** 2026-04-01

**Authors:** Mohamed Sherif Morsy, Nandini Avijit Dutta, Elsaid Ibrahim Eldessouky, Mamdouh Mahmoud Kabil, Hamdy Abd El Azim El-Koumy, Nehal Nailesh Mehta, Amr Lotfy Ali, Soumya Jena, Haochen Zhang, Dirk-Uwe Bartsch, Lingyun Cheng, Cheolhong An, Truong Nguyen, William R. Freeman

**Affiliations:** 1 Jacobs Retina Center, 9415 Campus Point Drive, La Jolla, CA 92093 USA; 2 Viterbi Family Department of Ophthalmology and Shiley Eye Institute, University of California, La Jolla, 92093 USA; 3 Ophthalmology Department, Faculty of Medicine, Tanta University, Tanta, Egypt; 4 National Institute of Technology, Tiruchirapalli, India; 5 Department of Electrical and Computer Engineering, University of California San Diego, La Jolla, CA USA

**Keywords:** Optical coherence tomography angiography, Artificial intelligence, Neovascular age-related macular degeneration, Treatment response, Anti-VEGF therapy, Paired imaging, Computational biology and bioinformatics, Diseases, Health care, Medical research

## Abstract

Artificial intelligence (AI) has shown promise in retinal imaging, yet its application to longitudinal optical coherence tomography angiography (OCTA) remains limited. This study developed and evaluated an AI model for classifying treatment response in neovascular age-related macular degeneration (nAMD) using paired OCTA images acquired before and after anti-VEGF therapy. In this retrospective cohort study, paired OCTA en-face images and corresponding OCT B-scans were collected for each treatment course. OCTA image pairs were manually segmented and aligned for AI input, while ground-truth labels (Improved, Unchanged, Worsened) were determined based on structural OCT findings and clinical visual acuity outcomes. After quality exclusion, 1033 OCTA pairs were included and divided into training, validation, and independent testing subsets. Two experienced retina specialists graded all OCTA pairs for comparison. On the test set, the AI model achieved an overall accuracy of 82.08%, with class-specific accuracies of 74.29% (worsened), 81.48% (unchanged), and 88.64% (improved). In contrast, overall human grading accuracy was 61.40%. Human graders were significantly more likely to misclassify treatment response than the AI model for all groups (odds ratio = 2.88; 95% CI 1.68–4.92; *p* < 0.0001). These findings demonstrate that AI-based paired OCTA analysis can provide a more accurate and objective assessment of treatment response in nAMD.

**Supplementary Information:**

The online version contains supplementary material available at 10.1038/s41598-026-42999-7.

## Introduction

Age-related macular degeneration (AMD) is a leading cause of irreversible visual impairment in developed countries, accounting for approximately 7% of blindness worldwide^1^. Choroidal neovascularization is the principal cause of vision loss in neovascular AMD (nAMD). Intravitreal anti-vascular endothelial growth factor (anti-VEGF) therapy remains the standard of care and has substantially improved both anatomical and visual outcomes^2^. Despite widespread adoption, real-world data show that a considerable proportion of eyes respond suboptimally, exhibiting persistent or recurrent fluid, pigment epithelial detachment (PED), or episodes of lesion reactivation^3^.

Fluorescein angiography (FA) has traditionally been the gold standard for detecting macular neovascularization (MNV), but it is invasive and requires intravenous dye injection. The introduction of optical coherence tomography (OCT) provided high-resolution, noninvasive cross-sectional retinal images, enabling precise assessment of disease severity and monitoring of treatment response. More recently, OCT angiography (OCTA) has allowed noninvasive visualization of the chorioretinal microvasculature, improving characterization of microvascular changes in nAMD and supporting earlier detection of neovascular activity for timely intervention^4–6^. We therefore hypothesized that changes in MNV morphology observed on OCTA correlate with clinical response during treatment and could serve as a valuable tool for guiding therapeutic decisions and evaluating the efficacy of current and emerging anti-VEGF therapies.

Several studies have compared OCTA with FA for detecting MNV in nAMD, reporting OCTA sensitivity of 66.7–86.5% and specificity of 67.6–100% when benchmarked against FA^5,7^. However, fluorescein angiography may not represent an ideal reference standard for neovascular detection, as it is primarily sensitive to vascular leakage rather than direct visualization of the neovascular structures themselves. Prior work has demonstrated that FA can underestimate or miss pathological vascular changes in other causes of retinal edema, even when structural OCT clearly shows intraretinal or subretinal fluid.^8^ This limitation suggests that FA-based comparisons may underestimate the true diagnostic capability of OCTA, which uniquely enables direct, depth-resolved visualization of pathological neovascular networks. Early morphological characterization of macular neovascularization on OCTA was described by El Ameen et al.^9^ and Kuehlewein et al.^10^, who identified distinct vascular patterns: a medusa pattern, featuring a central trunk with radially branching vessels; a sea-fan pattern, in which vessels extend predominantly along a single plane; and a glomerulus pattern, characterized by a compact network of tightly interconnected vessels. The authors also described an undefined MNV pattern, characterized by the absence of a discernible vascular configuration or by poorly visualized neovascular structures. Notably, they found no significant association between any OCTA-defined MNV pattern and clinical features such as pigment epithelial detachment (PED), subretinal hyperreflective material (SHRM), retinal pigment epithelium (RPE) atrophy, or the number of anti-VEGF injections administered.^10^.

Currently, decisions regarding anti-VEGF retreatment are guided by the presence of intraretinal fluid (IRF), subretinal fluid (SRF), PED, or hemorrhage as markers of active MNV growth. The mere detection of MNV on OCTA does not automatically indicate the need for treatment, as some lesions may be non-exudative and stable for years. Even exudative MNV can persist on OCTA after successful therapy in a temporarily inactive state. Reliable assessment of MNV activity on OCTA could enable better detection and classification of active disease and timely intervention before irreversible retinal damage occurs^11^.

Recent OCTA-based studies suggest that microvascular biomarkers may correlate with clinical activity. For instance, a study evaluating type 1 MNV demonstrated that eyes with a higher number of open-ended peripheral vessels on OCTA were more likely to experience earlier recurrence of exudation under a PRN anti-VEGF regimen. This highlights the potential of OCTA-derived vascular features to reflect underlying disease activity^12^.

Large-scale validation of OCTA metrics requires automated analysis, and advances in artificial intelligence (AI), particularly deep learning, provide powerful tools for this purpose. Parallel progress in OCTA imaging and AI-driven analysis allows extraction of reproducible, clinically meaningful vascular information.^13^ Deep learning has been increasingly applied to ocular imaging, especially fundus photography and OCT, with AMD being a major focus^14^. Contemporary AI applications in OCTA include accurate detection of pathologies such as CNV, precise quantification of retinal perfusion, and reliable disease diagnosis^15^. Our group previously developed an AI model that classified CNV stages on 2D OCTA images, achieving higher accuracy than human experts in distinguishing normal, dry AMD, active wet AMD, and remission cases^16^.

Recently, Wongchaisuwat et al.^17^ applied a deep-learning algorithm to combined OCT and OCTA scans and demonstrated high sensitivity (96.4%) and specificity (98.3%) in detecting macular neovascularization in exudative AMD. However, as structural OCT features were included, the independent contribution of OCTA to model performance remains uncertain, highlighting the need for OCTA-focused AI analysis.

To date, most artificial intelligence applications involving OCT angiography in neovascular AMD have focused on cross-sectional image analysis, such as detection of macular neovascularization^16,17^, lesion characterization, or disease staging from single time-point scans^18^. Although several longitudinal OCTA studies, including prior work from our group, have described vascular changes following anti-VEGF therapy, these investigations have relied on manual or semi-quantitative human assessment of sequential images, often focusing on descriptive changes in lesion area, vessel density, or morphology^19^. As a result, longitudinal interpretation remains time-consuming, subjective, and prone to inter-observer variability. Recent work has demonstrated the value of paired image analysis using artificial intelligence, with a Siamese-network model successfully applying pre- and post-treatment structural OCT B-scans to assess response to anti-VEGF therapy^20^. This highlights the importance of longitudinal, paired imaging for capturing treatment-related changes; however, such approaches have been limited to structural OCT and have not been extended to OCT angiography, where vascular remodeling rather than fluid dynamics is the primary signal of disease activity.Given that clinical decision-making in neovascular AMD is inherently longitudinal and depends on comparison of imaging findings over time, an AI framework capable of directly analyzing paired OCTA scans could provide objective assessment of treatment-related vascular remodeling, reduce observer variability, and support more consistent evaluation of therapeutic response.

Building on this work, the aim of the current study was to develop an AI model capable of characterizing treatment response using paired OCTA scans, with each unit of analysis representing a treatment course defined by pre- and post-treatment images from the same eye. The three response categories used in this study, improved, unchanged, and worsened, were deliberately chosen because they capture the most clinically meaningful aspects of disease activity. These categories align with how retina specialists assess treatment success, as they correspond to changes in fluid status, need for retreatment, and expected visual outcomes. Classifying eyes according to these clinically grounded categories ensures that the AI model outputs information that is directly relevant to decision-making and patient care.

## Methods

This retrospective study was conducted at the Department of Ophthalmology, University of California San Diego (UCSD), Shiley Eye Institute, using patient data from January 1, 2022, to September 31, 2025. Data were obtained by analyzing patient records from our healthcare data entry electronic records (EPIC, Verona, Wisconsin, USA). Institutional Review Board approval was obtained from the University of California San Diego for review of patient charts and imaging data (IRB #120516). Due to the retrospective nature of the study, the requirement for informed consent was waived by the IRB. The study was conducted in accordance with the tenets of the Declaration of Helsinki and complied with the Health Insurance Portability and Accountability Act (HIPAA) regulations. All data were de-identified to ensure patient confidentiality.

Patients with neovascular AMD who underwent OCT angiography imaging before and after anti-VEGF treatment were included. For each treatment course, paired OCTA images consisting of one pre-treatment and one post-treatment scan acquired from the same eye were analyzed if they had a complete and correctly aligned pre- and post-treatment OCTA scan set and demonstrated a clear structural change on corresponding OCT B-scans with the age over 50, presence of AMD diagnosis, and the presence of high-quality OCTA scans in the database (defined as lack of motion artifacts and background noise and quality index Q had to be > or = 25). Exclusion criteria included minimal or ambiguous structural change on OCT, poor image quality, significant artifacts, insufficient alignment between paired OCTA scans, or media opacity.A total of 1052 OCTA image pairs were initially identified. 19 pairs were excluded due to poor alignment, hazy media, or inadequate image quality. The final dataset therefore consisted of 1033 analyzable OCTA pairs. A total of 256 eyes from 220 patients were included in the analysis with some eyes contributing multiple treatment courses over time. Anti-VEGF therapy was administered according to routine clinical practice, typically beginning with a loading phase followed by a treat-and-extend or pro re nata regimen at the discretion of the treating physician. Each treatment course was analyzed independently and defined as a discrete therapeutic interval with a clearly defined start and end point, corresponding to a change in treatment status and resulting in a specific clinical outcome (improved, unchanged, or worsened) based on post-treatment evaluation. Treatment courses were not identical across patients or eyes and reflected routine clinical management of neovascular AMD. Treatment intervals, number of injections, and imaging timing varied according to individualized clinical decision-making.This design reflects routine longitudinal management in neovascular AMD.

All OCTA images were acquired using a Heidelberg Spectral-domain OCT system (Spectralis SD-OCT; Heidelberg Engineering, Heidelberg, Germany). The OCTA scan protocol consisted of a volumetric scan consisting of 512 B-scans over a 3 × 3 mm (10° × 10°) field of view, with a B-scan spacing of 6 μm and an automatic real-time (ART) image averaging of 5 frames.From each volumetric acquisition, four two-dimensional en-face OCTA projections corresponding to predefined retinal slab segmentations were generated by the device software. No fixed or predefined time interval was imposed between pre- and post-treatment OCTA acquisitions. Pre-treatment OCTA was obtained prior to anti-VEGF injection, and post-treatment OCTA was acquired at the subsequent follow-up visit used for clinical assessment of treatment response. Accordingly, the interval between pre- and post-treatment OCTA acquisitions varied based on individualized clinical management, reflecting real-world longitudinal care in neovascular AMD, with a mean follow-up interval of 46 ± 36 weeks.

### Data collection and imaging acquisition

For each treatment course, two imaging modalities were obtained: (1) OCTA en-face images for AI model input and (2) OCT B-scans for establishing ground-truth labels. OCTA images were exported as uncompressed TIFF files from the avascular retina slab. All images were manually segmented to ensure consistent slab positioning and were carefully aligned to match the scan pattern between pre- and post-treatment acquisitions. Manual slab segmentation was performed using a standardized protocol to ensure consistent slab positioning between pre- and post-treatment OCTA acquisitions, including in cases with intraretinal fluid or pigment epithelial detachment. To support the reliability of this process, representative examples demonstrating slab positioning on OCT B-scans and the corresponding en-face OCTA images are provided in the Supplementary Material (Supplementary Figs. S2–S4), including both simple and complex cases. OCT B-scans were reviewed independently from the OCTA images and were used solely to assess treatment response based on structural changes, including variation in intraretinal or subretinal fluid, alterations in pigment epithelial detachment (PED), and clinically documented changes in best-corrected visual acuity (BCVA). All imaging was reviewed on high-resolution monitors to ensure optimal visualization of choroidal neovascularization morphology.

### Dataset preparation for model development

Each paired OCTA treatment course was assigned a single treatment-response label (improved, unchanged, or worsened). Ground-truth labels were determined by expert review of paired OCT B-scans, with supportive consideration of best-corrected visual acuity. Classification followed predefined qualitative criteria rather than a quantitative scoring system. Improved was defined as resolution or substantial reduction of intraretinal and/or subretinal fluid and/or improvement in pigment epithelial detachment morphology on OCT B-scans, with stable or improved visual acuity. Worsened was defined as new or increased intraretinal or subretinal fluid and/or progression of pigment epithelial detachment morphology, with stable or decreased visual acuity. Unchanged was defined as the absence of meaningful structural change on OCT B-scans, regardless of persistent neovascular structures visible on OCTA, with stable visual acuity.Structural OCT findings were considered the primary determinant of treatment response, while visual acuity served as a supportive clinical measure and did not override structural imaging findings. OCTA images were not used to assign ground-truth labels. Each image pair was assigned a numeric code: 0 (Worsened), 1 (Unchanged), or 2 (Improved). Labels were stored in a master CSV file linking the filenames of each pre- and post-treatment OCTA image to its assigned category. For the AI training, we followed the standard training/validation/testing data split protocol; The final dataset of 1,033 labeled image pairs was randomly divided into: training set with 800 pairs, validation set with 127 pairs and testing set with 106 pairs. Each sample consisted of paired pre- and post-treatment OCTA en face images from the same eye, including both the avascular retina slab and the choriocapillaris slab, aligned to identical scan patterns. In the training set (*n* = 800), 200 cases were classified as worsened, 290 as unchanged, and 310 as improved. The validation set (*n* = 127) included 32 worsened, 41 unchanged, and 54 improved cases. The independent test set (*n* = 106) comprised 35 worsened, 27 unchanged, and 44 improved cases, reflecting a balanced representation of all three treatment-response categories.

### AI method

**Fig. 1:**
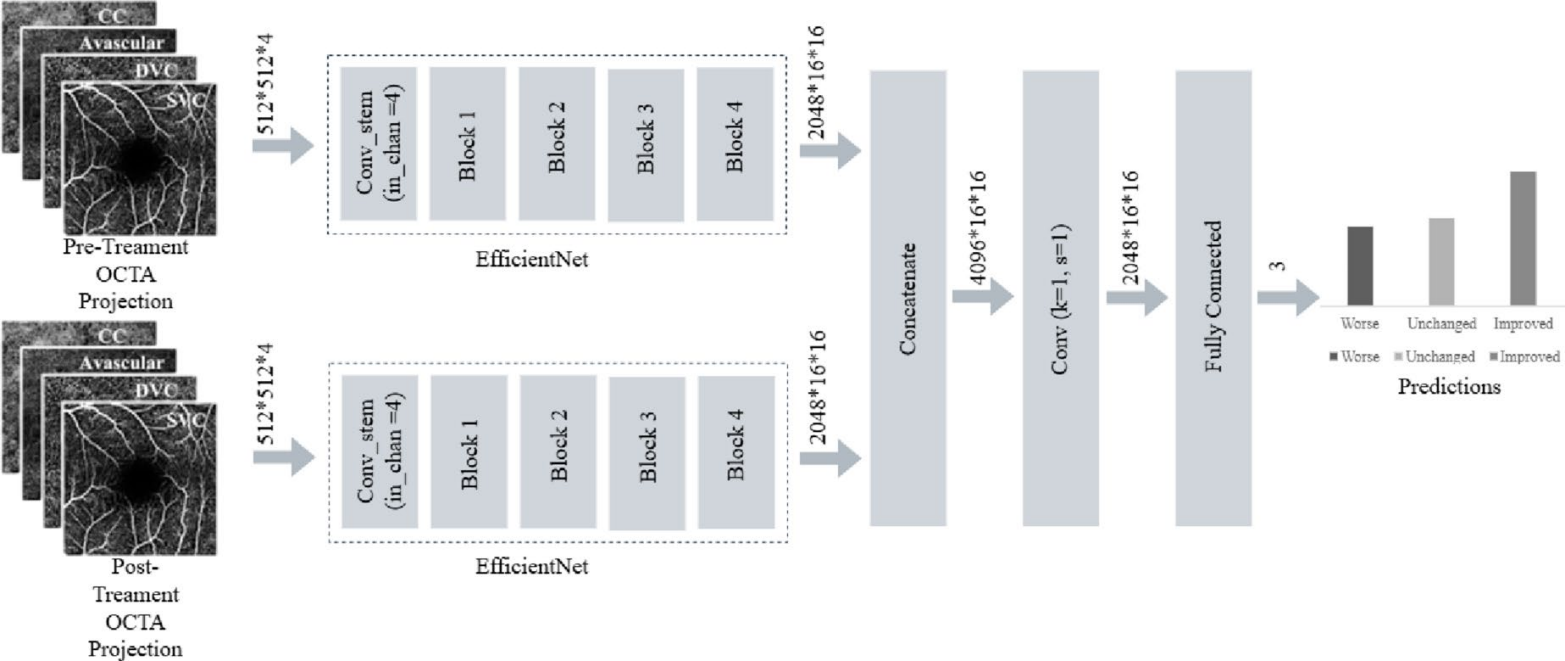
The architecture of the classifier employed in our experiments is illustrated here. The model consists of two parallel EfficientNet-B5 backbones with minor modifications. Each branch processes a four-channel OCTA input composed of CC, avascular, DVC, and SVC projections through a modified convolutional stem to accommodate the four channels of input OCTA projections. Feature representations extracted from the two branches are concatenated and fused using an additional convolution, followed by a fully connected layer that outputs predictions for three classification categories.

For AI training, we used a standard 800/127/106 split for the training, validation, and test sets, with the same test set also used for comparison with human graders. The model is trained end-to-end from scratch specifically for OCTA-based treatment-response prediction. As depicted in Fig. 1, the network is built on a dual-branch EfficientNet backbone, modified to accept the four en-face OCTA projections—SVC (superficial vascular complex), DVC (deep vascular complex), avascular complex, and CC (choriocapillaris)—as a four-channel input. Because standard EfficientNet models are designed for three-channel RGB images, the input stem was adjusted to accommodate four channels. Separate EfficientNet-B5 encoders process the pre-treatment and post-treatment OCTA scans through the full sequence of EfficientNet layers, producing high-level feature maps of size 2048 × 16 × 16. These two feature maps are concatenated to form a 4096-channel representation and subsequently compressed via a 1 × 1 fusion convolution, after which global average pooling and a fully connected layer generate the final three-class prediction corresponding to improved, unchanged, or worsened treatment response.

Before being fed into the model, OCTA projections underwent intensity scaling along with simple spatial augmentations, including random flipping and random rotation, to enhance robustness and increase the diversity of the training set. The network was trained using a batch size of 8 and optimized using the Adam optimizer with a learning rate of 1 × 10^−4^ and an L2 regularization term of 1 × 10^−5^. To promote stable learning across epochs, a StepLR scheduler was used to reduce the learning rate by a factor of 0.5 every 20 epochs, allowing the model to shift smoothly from coarse updates in early training to finer adjustments later on. Cross-entropy loss served as the training objective, and the model achieving the highest validation accuracy during training was retained as the final classifier. Evaluation was performed on the held-out test set using accuracy, confusion matrix, and detailed classification metrics.A flowchart summarizing OCTA image-pair inclusion, exclusion criteria, and dataset allocation is provided in Supplementary Fig. S1.

Dataset splitting was performed at the paired OCTA treatment-course level, rather than at the eye or patient level. Each treatment course consisted of a unique pre- and post-treatment OCTA image pair and was included only once in either the training, validation, or test set. No identical OCTA image pairs were shared across datasets.Because neovascular AMD requires longitudinal follow-up, multiple treatment courses from the same eye or patient, obtained at different time points and representing distinct therapeutic responses, were permitted to appear in different datasets. All image pairs were temporally distinct, and no overlapping pre- or post-treatment images were used across datasets. Eyes contributing multiple treatment courses typically represented cases with recurrent or complex disease rather than straightforward responders; no weighting or oversampling based on patient or eye frequency was applied during model training.

### Human grading and reliability assessment

Two retina specialists with extensive experience in OCTA interpretation (AA and NM) independently reviewed all included image pairs. Each grader had more than 6 years of clinical experience and routinely interpreted OCT and OCTA imaging in the management of neovascular age-related macular degeneration. They evaluated the same en-face OCTA slab images that were used as input to the EfficientNet model. Uncompressed OCTA TIFF files from the avascular slab were displayed side-by-side on high-resolution monitors. No adjudication or consensus grading was performed. For comparison between AI and human performance, each grader’s classification was treated as an independent human judgment. Classifications were evaluated against the ground-truth treatment-response label derived from OCT B-scans and clinical outcomes. Human performance was therefore analyzed using a pooled (stacked) approach, reflecting real-world inter-grader variability in OCTA interpretation.This strategy was chosen to capture inter-grader variability and to reflect routine clinical interpretation of OCTA images in real-world practice.

### Statistical methods

for human experts grading data, data from the two graders were analyzed separately along with AI classified data against the ground truth classification. When the grader’s classification agrees to the ground truth, the grading was coded as the “Hit”, otherwise coded as “Miss”. A contingency table along with Chi square assessment was used to evaluate the accuracy of the human experts’ classification and AI classification. In addition, an analysis of means for proportions was performed to examine the grading accuracy by true classification levels using a critical significance level of 0.05 (Type I error). For overall comparison among human graders and AI, a generalized linear mixed model was performed using image ID as a random effect. Subsequent post hoc tests were conducted using Student’s t All Pairwise Comparisons. JMP^®^ version 18 (2025) (JMP Statistical Discovery LLC, Cary, NC, USA) was used. Classification performance was evaluated using accuracy, defined as the proportion of treatment courses correctly classified across the three response categories (improved, unchanged, worsened), incorporating both false-positive and false-negative predictions.

## Results

A total of 1052 paired OCTA scans were initially identified. Based on ground-truth labeling from OCT B-scans, 426 pairs (42.6%) were classified as Improved, 358 pairs (35.8%) as Unchanged, and 268 pairs (26.8%) as Worsened. Each pair was from a clinically identified treatment course with a clear end result. After excluding 19 pairs due to poor alignment, media opacity, or inadequate image quality, 1033 paired OCTA scans were included in the final analysis.The mean age of participants was 81.21 ± 9.25 years. Females represented 53.3% of the cohort, while males accounted for 46.7%. Regarding lens status, 31.11% of eyes were phakic without visually significant cataract, and 68.89% were pseudophakic.

### AI model performance

**Fig. 2:**
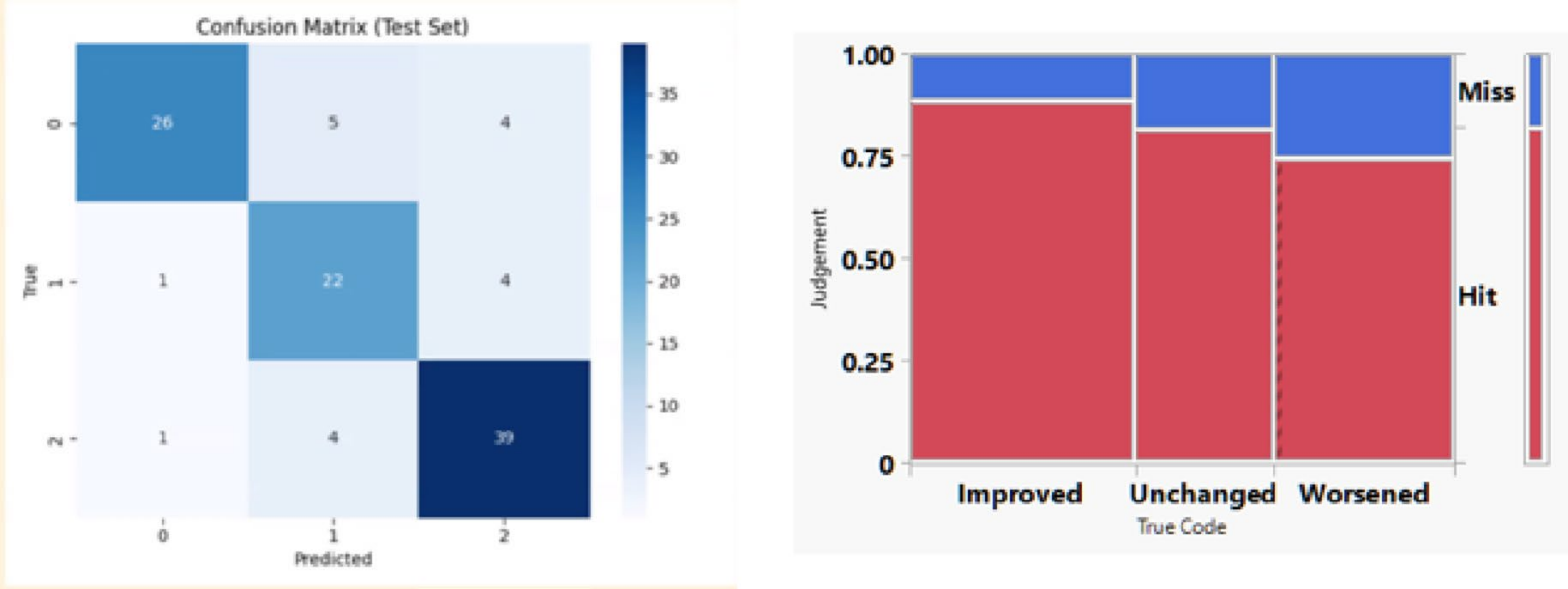
Contingency analysis and Confusion Matrix of the AI model across the three treatment response categories.

The deep learning model demonstrated strong overall ability to classify treatment response using paired OCTA images. On the independent test set, the model achieved an overall accuracy of 82.08%. Class-specific performance derived from the confusion matrix showed: for the Worsened group 74.29% correctly classified, for the Unchanged group : 81.48% correctly classified, for the Improved group : 88.64% correctly classified. The model performed best in identifying improved cases, followed by unchanged, while worse cases showed the greatest overlap with the adjacent classes (Fig. 2)

**Fig. 3:**
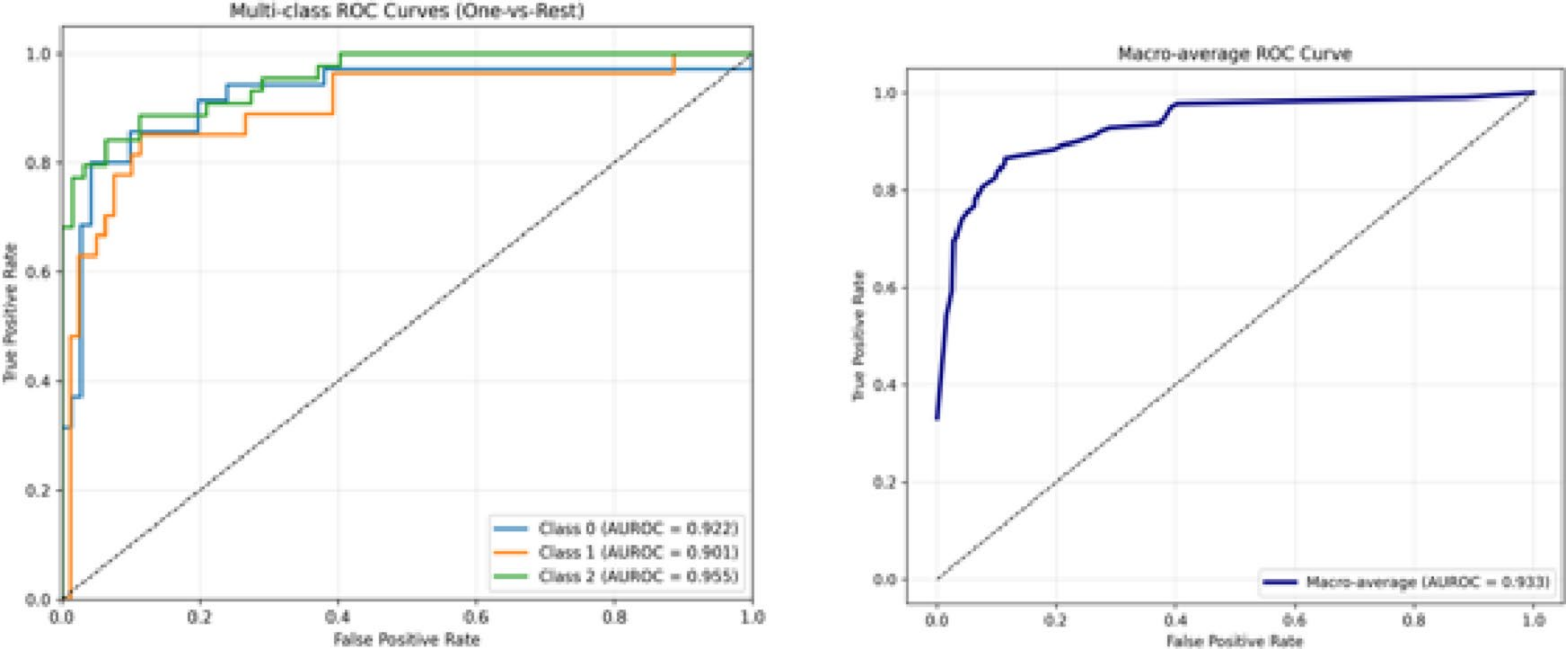
One-vs-rest (OvR) receiver operating characteristic (ROC) curves for multiclass classification of treatment response using the AI model. ROC curves are shown for each treatment-response category (worsened, unchanged, improved), along with the macro-averaged ROC curve representing overall multiclass discrimination performance.

Multiclass discrimination performance was additionally assessed using a one-vs-rest (OvR) AUROC analysis, yielding a macro-averaged AUROC of 0.926, with class-specific AUROC values of 0.922 (worsened), 0.901 (unchanged), and 0.955 (improved) (Fig. 3).

### Human grading performance

**Fig. 4:**
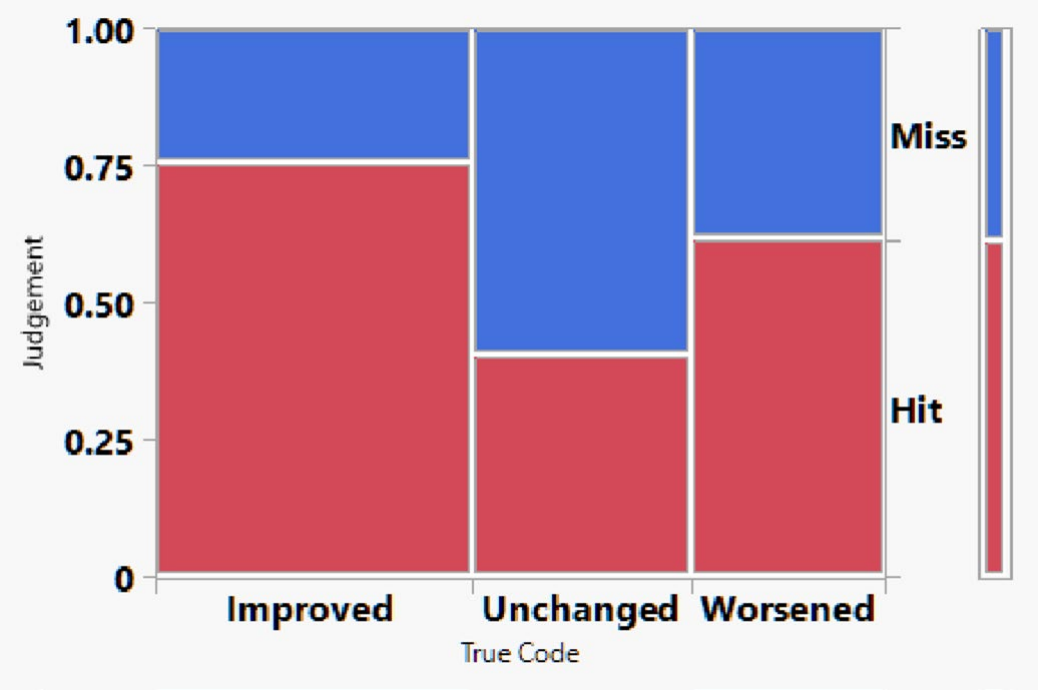
Contingency analysis of classification performance for the overall human grader across the three treatment-response categories (worsened, unchanged, and improved).

**Fig. 5:**
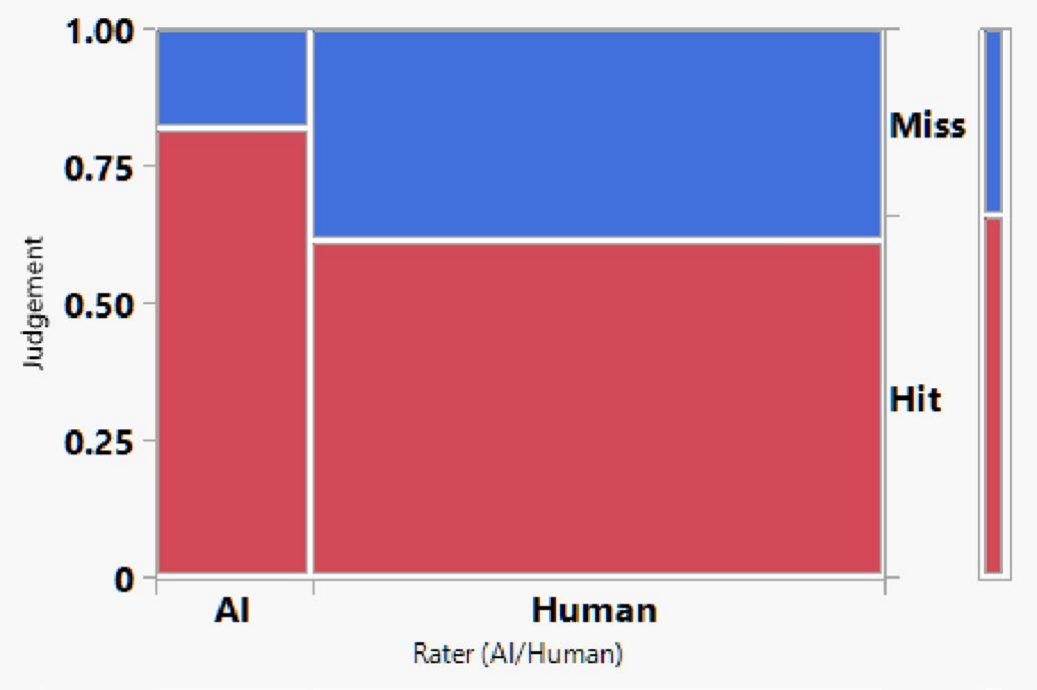
Contingency stacked analysis comparing classification performance of the AI model versus the overall human grader across the three treatment-response categories (worsened, unchanged, and improved).

Two retina specialists independently evaluated all OCTA image pairs without access to OCT B-scans or clinical information. Contingency analysis using stacked hit/miss plots showed that the human performance remained inconsistent across categories with an overall human accuracy of 61.40%, substantially lower than the AI model. Class-specific human performance showed: for the Worsened group : 61.76% correctly classified. For the Unchanged group: 40.52% correctly classified. For the Improved group: 75.60% correctly classified. Among those three categories, the accuracy for the Unchanged cases was significantly poorer (*p* < 0.05) while significantly better for the improved cases (*p* < 0.05, Analysis of Means for Proportions). Human graders demonstrated the greatest difficulty identifying unchanged cases. (Table 1). Inter-grader variability was observed across treatment-response categories and is summarized in Table 1, highlighting the subjective nature of OCTA-based assessment (Figs. 4 and 5).

### Comparison between AI and human graders

**Fig. 6:**
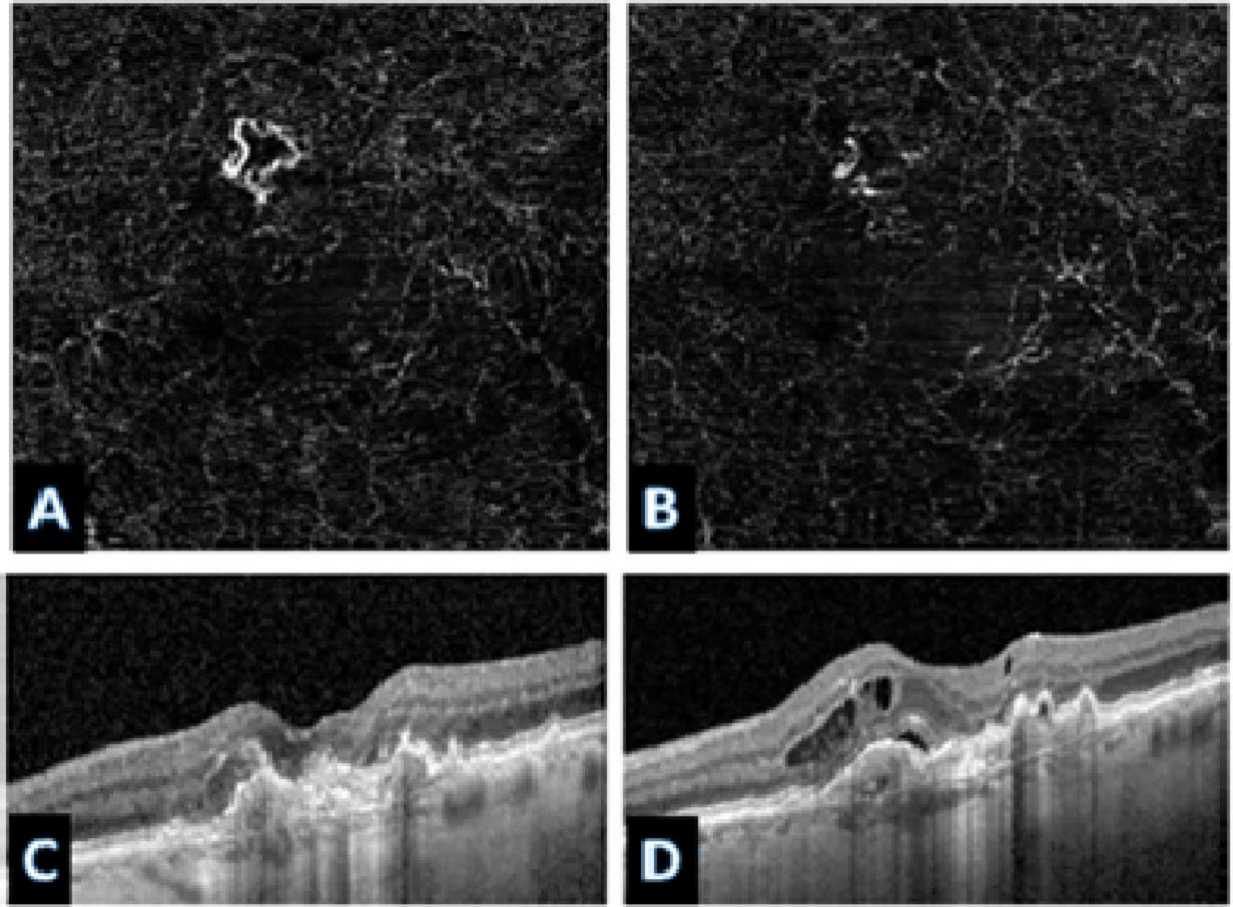
Representative case correctly classified by the AI model but misclassified by human graders. This example shows a worsened treatment response accurately identified by the AI model. Pre- and post-treatment OCTA images (**A**,**B**) display subtle vascular changes that led human graders to incorrectly label the case as improved. In contrast, the corresponding structural OCT images (**C**,**D**) confirm clear worsening, establishing the ground truth. This case illustrates the AI model’s ability to detect complex or non-intuitive disease patterns that may be visually underappreciated by human experts.

**Fig. 7:**
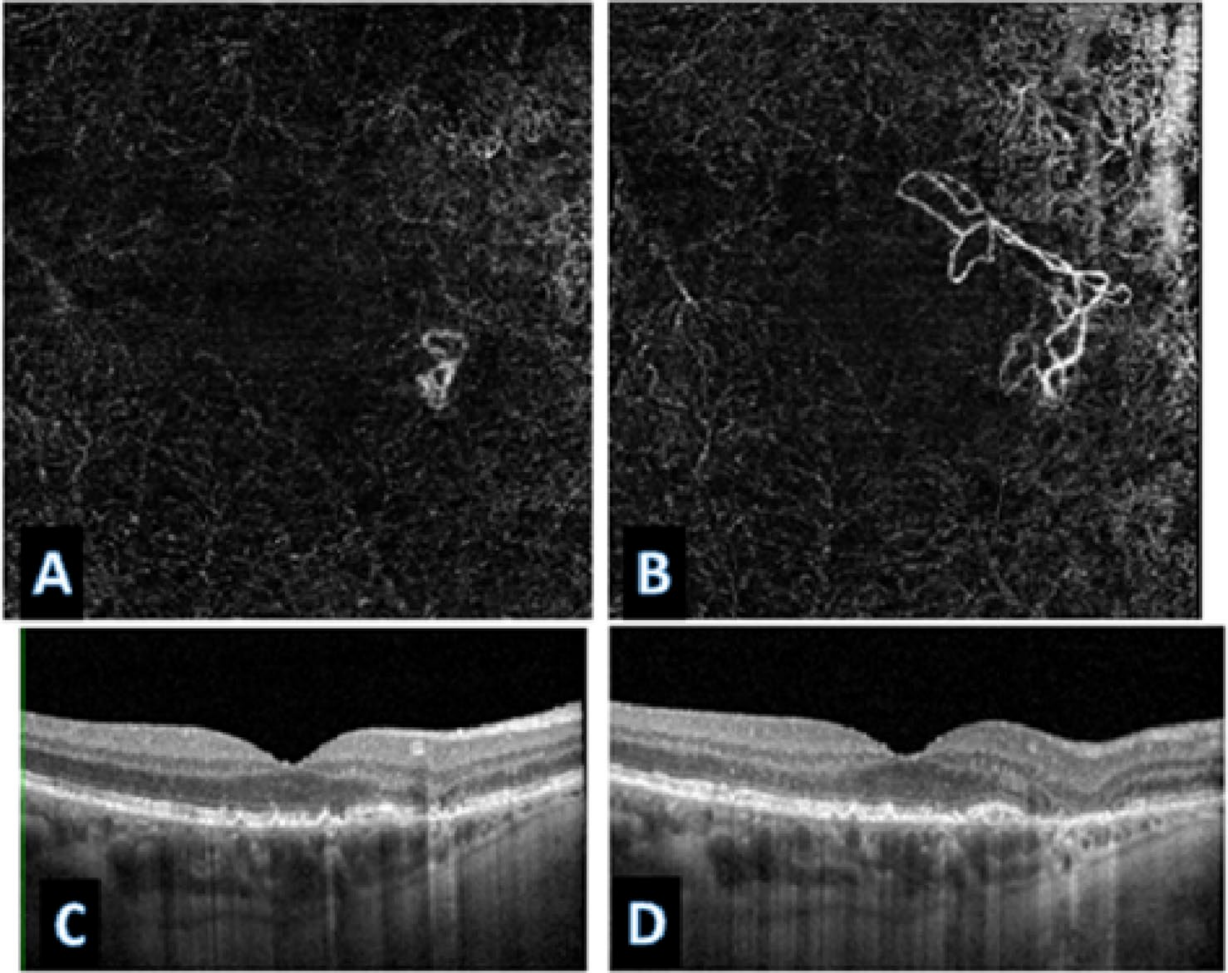
Representative case correctly classified by the AI model but misclassified by human graders (unchanged status). Post-treatment OCTA showed a more prominent CNV appearance, leading human graders to overinterpret vascular changes and incorrectly label the case as worsened. Structural OCT images (**C** and **D**), however, demonstrated no new or residual fluid, despite some extrafoveal atrophy, confirming an unchanged clinical status. The AI model correctly identified the case as unchanged, reflecting its ability to recognize stable textural and geometric patterns that may be visually misleading on OCTA.

**Fig. 8:**
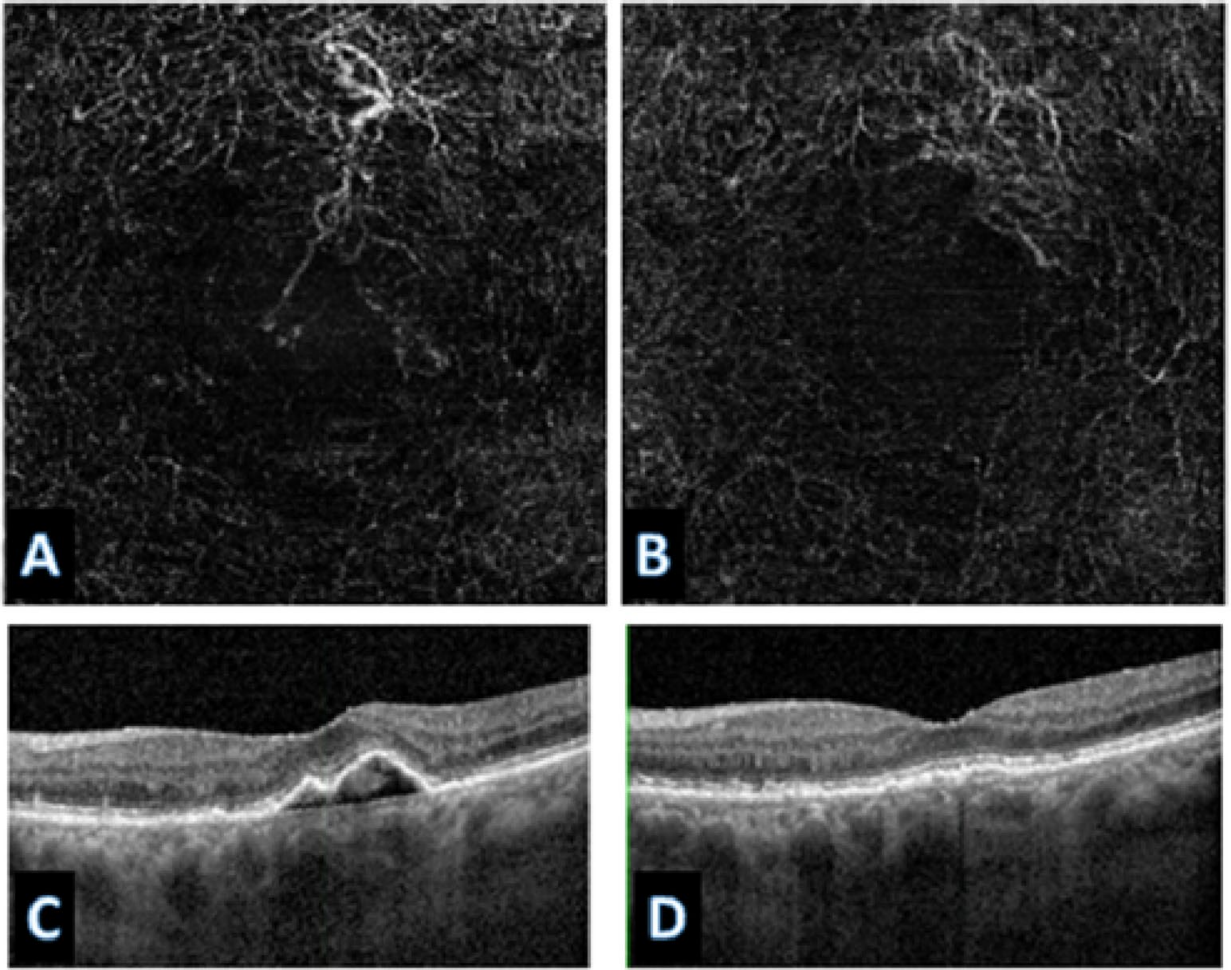
Representative case correctly classified by both the AI model and human graders (improved status).In this example, pre- and post-treatment OCTA images (**A**,**B**) demonstrate clear and easily recognizable vascular improvement. Structural OCT images (**C**,**D**) confirm resolution of fluid, establishing the ground truth. This case illustrates a straightforward scenario in which both human graders and the AI model converged on the correct classification.

**Fig. 9:**
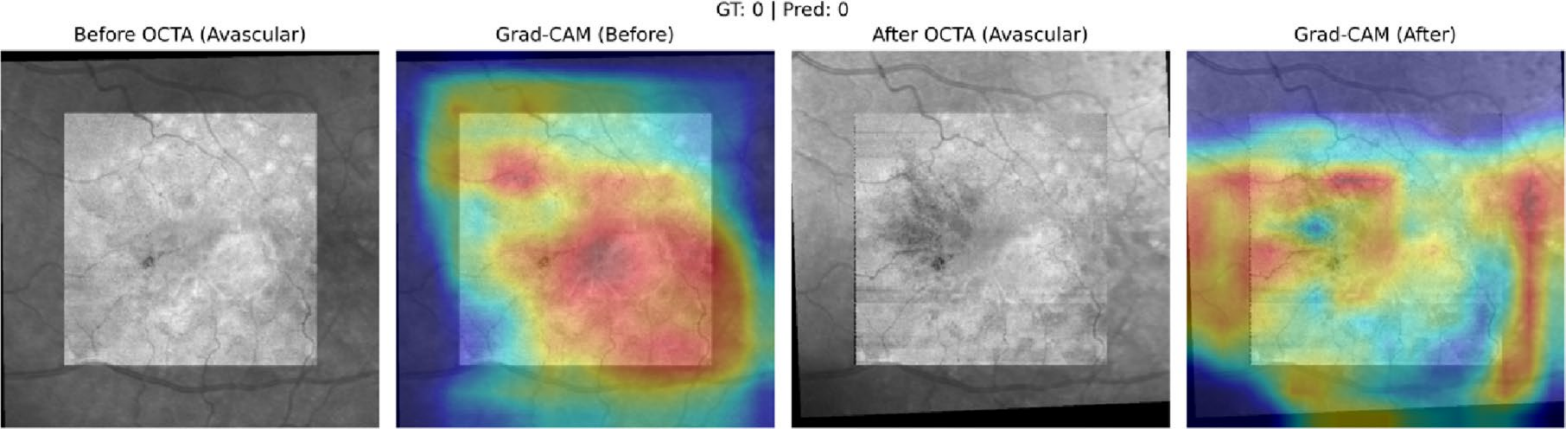
Saliency heatmap for the AI model corresponding to the worsened-treatment example shown in Fig. 6. Warmer colors indicate regions with greater contribution to the model’s prediction based on paired OCTA inputs. Gradient-weighted Class Activation Mapping (Grad-CAM) saliency heatmaps generated using PyTorch (version 1.11.0; https://pytorch.org). Saliency maps were computed from the final convolutional layers of the dual EfficientNet encoders, resized using OpenCV (version 4.6.0; https://opencv.org), and visualized with Matplotlib (version 3.5.2; https://matplotlib.org). Heatmaps are projected onto the avascular OCTA slab.

**Fig. 10:**
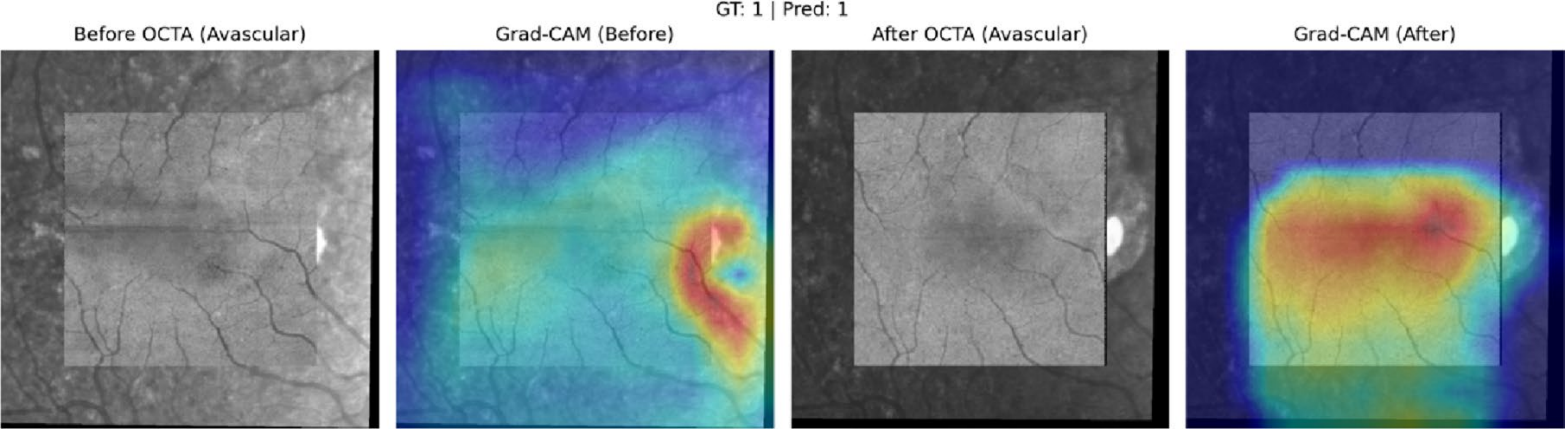
Saliency heatmap for the AI model corresponding to the Unchanged-treatment course example shown in Fig. 7. Warmer colors indicate regions with greater contribution to the model’s prediction based on paired OCTA inputs. Gradient-weighted Class Activation Mapping (Grad-CAM) saliency heatmaps generated using PyTorch (version 1.11.0; https://pytorch.org). Saliency maps were computed from the final convolutional layers of the dual EfficientNet encoders, resized using OpenCV (version 4.6.0; https://opencv.org), and visualized with Matplotlib (version 3.5.2; https://matplotlib.org). Heatmaps are projected onto the avascular OCTA slab.

**Fig. 11:**
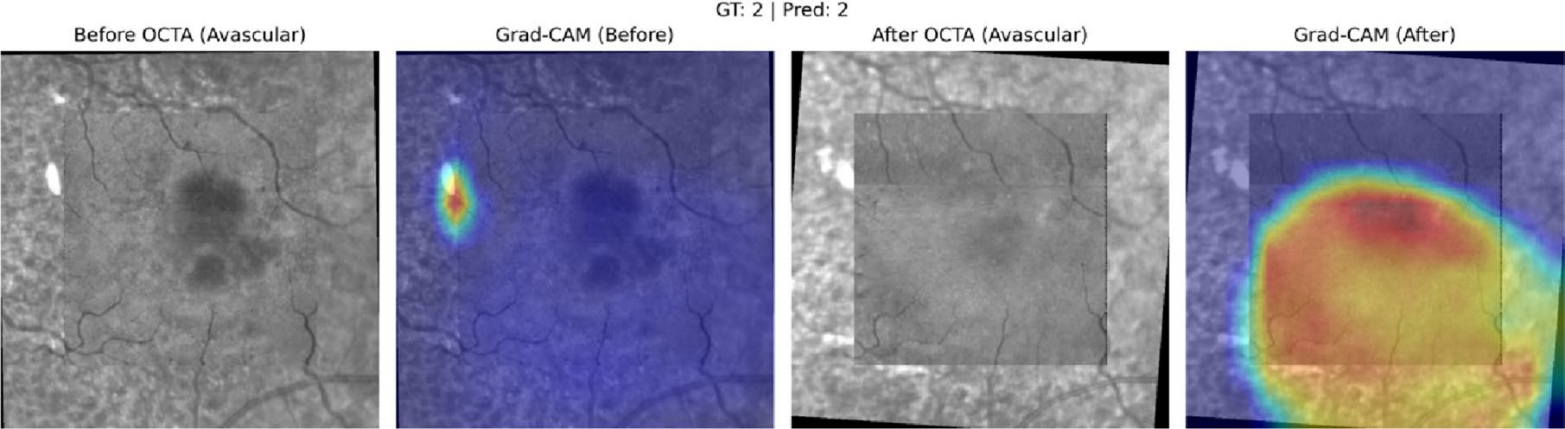
Saliency heatmap for the AI model corresponding to the Improved-treatment course example shown in Fig. 8. Warmer colors indicate regions with greater contribution to the model’s prediction based on paired OCTA inputs. Gradient-weighted Class Activation Mapping (Grad-CAM) saliency heatmaps generated using PyTorch (version 1.11.0; https://pytorch.org). Saliency maps were computed from the final convolutional layers of the dual EfficientNet encoders, resized using OpenCV (version 4.6.0; https://opencv.org), and visualized with Matplotlib (version 3.5.2; https://matplotlib.org). Heatmaps are projected onto the avascular OCTA slab.

Across all treatment response classes, the AI model outperformed human graders when assessing paired OCTA images alone. The model demonstrated a 20.6% point improvement in overall accuracy compared with experts (82% vs. 61.4%). The AI model achieved markedly higher correct classification rates. (*p* < 0.001). A detailed comparison of accuracy across evaluators is provided in Table 1, which presents category-specific and overall accuracies for the AI model and the combined stacked human analysis. Figures 6 and 7, and 8 present representative examples across the three treatment-response categories, illustrating cases correctly identified by both the AI model and human graders, as well as cases in which the AI model classified the response accurately while human graders misclassified the status. Saliency heatmaps corresponding to these representative cases were generated to visualize regions contributing to the AI model’s predictions and are shown in Figs. 9, 10 and 11. Statistical analysis demonstrated a significantly higher misclassification rate among human graders compared with the AI model. A likelihood ratio test (χ^2^ = 17.17, *p* < 0.0001) and Pearson chi-square test (χ^2^ = 15.81, *p* < 0.0001) confirmed a strong difference in error rates between raters. Fisher’s exact test further showed that human graders were significantly more likely to misclassify cases than the AI model (right-sided *p* < 0.0001). The odds ratio of misclassification was 2.88 (95% CI 1.68–4.92), indicating that humans were nearly three times more likely to produce an incorrect judgment than the AI.

To assess potential subject-level bias, an additional eye-level subanalysis was performed using the independent test set, in which only one eye was included. This eye-level test set consisted of 49 eyes for the AI model, yielding an overall accuracy of 73.47%. An analogous eye-level analysis was conducted for human graders, resulting in an overall accuracy of 58.20%. Although reduced sample size limited statistical power, AI performance remained numerically superior to human grading at the eye level, supporting the robustness of the primary treatment-course–level findings.(*p* = 0.06).

**Table 1:** Classification accuracy of the AI model and human graders across treatment response categories. This table reports the classification accuracy (%) for each treatment response category, Worsened, Unchanged, and Improved, as well as the overall accuracy for all evaluators. Accuracy metrics are presented for: the AI model, Human Grader 1, Human Grader 2, and the Combined Stacked Human Analysis (aggregated performance of both graders). The table enables direct comparison of performance across evaluators and response categories.

		Worsened group	Unchanged group	Improved group	Overall value	P value
Accuracy	AI model	74.29%	81.48%	88.64%	82.08%	
	Human grader 1	43.14%	48.28%	69.05%	55.96%	< 0.001*
	Human grader 2	80.39%	32.76%	82.14%	66.84%	0.004*
	Overall human	61.76%	40.52%	75.60%	61.40%	< 0.001*

*The p values referring to the comparison with AI model.

## Discussion

In this study, we developed and validated an AI model to assess treatment response in neovascular AMD using paired OCTA scans. The model demonstrated an overall accuracy of 82%, outperforming experienced human graders, who achieved 61.4% accuracy. Subgroup analysis showed the AI model performed particularly the best in identifying improved (87.7%) cases, whereas human graders showed lower accuracy, particularly for unchanged cases (40.5%). These findings suggest that AI-based evaluation of paired OCTA can provide more reliable and objective assessment of treatment response than conventional human grading.

A key finding of the present study is the substantial inter-grader and intra-grader variability observed in human interpretation of OCTA images, underscoring the subjective nature of vascular assessment. Human Grader 1 performed well in identifying worsened and improved cases but showed markedly reduced accuracy for unchanged cases, a category where OCTA changes are often subtle or counterintuitive. Human Grader 2 demonstrated more consistent but generally inferior performance across all groups, highlighting inter-grader variability observed in this study.Importantly, this variability persisted even when the graders’ results were considered together, indicating that aggregating human assessments does not overcome the inherent challenges of OCTA interpretation. In contrast, the AI model exhibited uniformly high and stable performance across all three clinically meaningful categories; worsened, unchanged, and improved. Unlike human graders, whose classification decisions can be disproportionately influenced by prominent vascular features (e.g., larger post-treatment CNV patterns that may not correlate with clinical activity), the AI leveraged global textural and geometric features, enabling it to correctly identify unchanged cases that were frequently misclassified by humans. This consistency suggests that AI may reduce the subjectivity and variability associated with OCTA interpretation, particularly in borderline or ambiguous cases where structural OCT shows stability (e.g., absence of fluid despite changes in lesion appearance).These findings reinforce the potential role of AI as a standardized decision-support tool capable of providing reliable, reproducible assessment of treatment response, especially in scenarios where human graders disagree or overinterpret OCTA signal fluctuations.

Previous studies have demonstrated the potential of AI in analyzing OCT and OCTA images for AMD, primarily for disease classification or CNV staging. For example,

Our group previously developed an AI model to stage CNV on 2D OCTA, classifying scans as normal, dry AMD, active wet AMD, or remission. The model achieved an overall accuracy of 80.4%, outperforming human experts, particularly in distinguishing active and remission cases, demonstrating the potential of AI to provide objective and reliable CNV grading^16^.

Most existing AI-based studies involving OCT angiography have focused on cross-sectional image analysis, addressing tasks such as macular neovascularization detection, lesion characterization, or disease classification from single time-point scans. In parallel, longitudinal OCTA studies have demonstrated that CNV lesions undergo pruning and remodeling over the course of treatment, with changes in vessel density, area, and complexity correlating with fluid resolution and visual outcomes^19,21,22^. However, these longitudinal studies have relied on manual or semi-quantitative assessment and did not incorporate AI-based analysis, leaving evaluation largely subjective and labor-intensive. By using paired pre- and post-treatment OCTA scans in our study, we directly address this gap. Our approach enables objective, quantitative evaluation of vascular remodeling over the course of therapy, a methodology that more closely mirrors clinical decision-making and reduces the subjectivity inherent in human grading.

An important observation in this study is the variability in human interpretation of OCTA images when assessing treatment response. Unlike structural OCT, which benefits from relatively standardized interpretation criteria centered on fluid and retinal morphology, OCT angiography lacks universally accepted guidelines for defining lesion activity or treatment response. Interpretation of OCTA is therefore inherently subjective, with clinicians often relying on individual experience, visual heuristics, and personal thresholds for what constitutes meaningful vascular change. As a result, disagreement among human experts is common when evaluating OCTA images, particularly in cases where vascular changes are subtle or counterintuitive.In the present study, this inter-grader variability was reflected in the differing accuracy profiles observed across treatment-response categories, as shown in the Results. Importantly, the goal of this work was to benchmark AI performance against routine human interpretation of OCTA images as encountered in clinical practice. For this reason, human performance variability was captured through category-wise accuracy analysis, highlighting the challenges of consistent OCTA interpretation and underscoring the potential value of AI-based approaches for providing more standardized and reproducible assessments.

Our findings demonstrate that AI-based evaluation of paired OCTA scans can reliably characterize treatment response in nAMD, outperforming experienced human graders. This objective assessment of macular neovascularization may provide insights into vascular remodeling and fluid resolution and could support more precise and timely treatment decisions, prognosticate future therapeutic needs and help guide which eyes respond best to which medications and even be used in clinical trials to develop better drugs. Clinically, such AI models have the potential to reduce subjectivity in monitoring, optimize retreatment intervals, and improve patient outcomes by identifying both early improvement and signs of recurrence. Furthermore, automated analysis could facilitate large-scale, longitudinal monitoring in clinical trials or real-world practice, providing standardized metrics for treatment efficacy and disease progression. By translating OCTA data into actionable insights, AI may help clinicians move beyond traditional fluid-based criteria and integrate vascular dynamics into routine decision-making.

It is important to recognize that OCT angiography primarily provides morphological information and does not directly depict vascular leakage or exudative activity, which remain central to clinical decision-making in neovascular AMD. Consequently, interpretation of OCTA images, particularly when performed visually by human graders. is inherently challenging, and reduced human performance in certain categories, especially unchanged disease, is not unexpected.Within this context, the key contribution of the present study is not simply that the AI model outperformed human graders, but rather that meaningful and clinically relevant treatment-response classification can be achieved from OCTA data alone despite these intrinsic limitations. By analyzing paired pre- and post-treatment OCTA scans, the AI model appears capable of capturing subtle, longitudinal morphological patterns associated with disease stability or response that may be difficult to consistently appreciate through human visual inspection. These findings suggest that AI-based OCTA analysis has the potential to complement conventional structural OCT by providing objective and reproducible insights into vascular remodeling, thereby enhancing treatment monitoring even in the absence of direct leakage information.

This study has several strengths. First, it leverages paired pre- and post-treatment OCTA scans, allowing direct assessment of treatment response rather than relying on cross-sectional imaging alone. Second, the AI model provides objective, reproducible quantification of vascular changes, outperforming human graders and reducing subjectivity. Third, the study includes a relatively large dataset, enhancing the generalizability of the findings.

However, certain limitations warrant consideration. First, dataset splitting was performed at the treatment-course level rather than strictly at the patient level. Because neovascular AMD requires longitudinal monitoring, multiple treatment courses from the same eye or patient, acquired at different time points and representing distinct clinical episodes, were permitted to appear in different dataset partitions. While this approach reflects real-world clinical practice, it may introduce correlated information across time points that could influence model performance. To address this concern, we performed an additional eye-level analysis in which each eye contributed only once to the independent test set, and the AI model continued to demonstrate superior performance compared with human graders.Secondly, The study is retrospective and based on high-quality OCTA scans, which may not fully represent real-world imaging variability. However we note that only a small percent of OCTA scans (1.8%) from the database were not used. Additionally, the AI model was trained on a single-center dataset, and external validation on independent populations and from multiple instruments is required.

Future studies should focus on external validation of AI models across multiple centers and imaging devices to ensure generalizability. Integrating multimodal data, including OCT B-scans, visual acuity, and other clinical parameters, could further enhance the predictive power of AI in monitoring treatment response. Additionally, prospective studies are needed to evaluate whether AI-guided management can improve clinical outcomes, optimize retreatment intervals, and reduce treatment burden.

In conclusion, our study demonstrates that AI-based analysis of paired OCTA scans can provide an accurate, objective assessment of treatment response in nAMD, outperforming conventional human grading. This approach represents a step toward more precise, data-driven management of neovascular AMD, with the potential to improve patient outcomes and support personalized therapy decisions.

## Supplementary Information

Below is the link to the electronic supplementary material.

Supplementary Material 1

Supplementary Material 2

Supplementary Material 3

Supplementary Material 4

## Data Availability

The data that support the findings of this study are not publicly available due to patient privacy considerations but are available from the corresponding author, Dr. William R. Freeman, upon reasonable request.

## References

[1] Bourne, R. R. A. et al. Causes of vision loss worldwide, 1990–2010: A systematic analysis. *Lancet Glob. Health*. **1**, (2013). 10.1016/S2214-109X(13)70113-X 25104599

[2] Baybora, H. Perifoveal retinal thickness changes after intravitreal aflibercept injection for choroidal neovascularization in age-related macular degeneration. *Photodiagn. Photodyn. Ther.* **46**, (2024). 10.1016/j.pdpdt.2024.104028 38438003

[3] Garweg, J. G. et al. Continued anti-VEGF treatment does not prevent recurrences in eyes with stable neovascular age-related macular degeneration using a treat-and-extend regimen: a retrospective case series. *Eye (Lond)*. **36**, 862–868 (2022). 33941877 10.1038/s41433-021-01562-6 PMC8956692

[4] Spaide, R. F. et al. Consensus nomenclature for reporting neovascular age-related macular degeneration data: Consensus on neovascular age-related macular degeneration nomenclature study group. *Ophthalmology*. **127**, 616–636 (2020). 31864668 10.1016/j.ophtha.2019.11.004 PMC11559632

[5] Faatz, H. & Lommatzsch, A. Overview of the use of optical coherence tomography angiography in neovascular age-related macular degeneration. *J. Clin. Med*. **13**, 5042 (2024). 10.3390/jcm13175042 PMC11396513 39274255

[6] Brown, D. M. et al. Ranibizumab versus verteporfin for neovascular age-related macular degeneration. *N. Engl. J. Med.* **355**, 1432–1444 (2006). 17021319 10.1056/NEJMoa062655

[7] Liang, M. C. et al. *Retina*. **36**, 2265–2273 (2016). 27285456 10.1097/IAE.0000000000001102

[8] Kozak, I. et al. Discrepancy between fluorescein angiography and optical coherence tomography in detection of macular disease. *Retina*. **28**, 538 (2008). 18398354 10.1097/IAE.0b013e318167270b PMC2666014

[9] El Ameen, A. et al. Type 2 neovascularization secondary to age-related macular degeneration imaged by optical coherence tomography angiography. *Retina*. **35**, 2212–2218 (2015). 26441269 10.1097/IAE.0000000000000773

[10] Kuehlewein, L. et al. Optical coherence tomography angiography of type 1 neovascularization in age-related macular degeneration. *Am. J. Ophthalmol.* **160**, 739–748e2 (2015). 26164826 10.1016/j.ajo.2015.06.030

[11] Faatz, H. & Lommatzsch, A. Overview of the use of optical coherence tomography angiography in neovascular age-related macular degeneration. *J. Clin. Med.*. **13**, 5042 (2024). 10.3390/jcm13175042 PMC11396513 39274255

[12] Choi, M., Kim, S. W., Yun, C., Oh, J. H. & Oh, J. Predictive role of optical coherence tomography angiography for exudation recurrence in patients with type 1 neovascular age-related macular degeneration treated with pro-re-nata protocol. *Eye* **37**, 34 (2022). 34992249 10.1038/s41433-021-01879-2 PMC9829809

[13] Hormel, T. T. et al. Artificial intelligence in OCT angiography. *Prog. Retin Eye Res.* **85**, (2021). 10.1016/j.preteyeres.2021.100965 PMC8455727 33766775

[14] Burlina, P. M. et al. Automated grading of age-related macular degeneration from color fundus images using deep convolutional neural networks. *JAMA Ophthalmol.* **135**, 1170–1176 (2017). 28973096 10.1001/jamaophthalmol.2017.3782 PMC5710387

[15] Hormel, T. T. et al. Artificial intelligence in OCT angiography. *Prog. Retin Eye Res.* **85**, (2021). 10.1016/j.preteyeres.2021.100965 PMC8455727 33766775

[16] Heinke, A. et al. Artificial intelligence for OCTA-based disease activity prediction in age-related macular degeneration. *Retina* **44**, 465 (2024). 37988102 10.1097/IAE.0000000000003977 PMC10922109

[17] Wongchaisuwat, N. et al. Detection of macular neovascularization in eyes presenting with macular edema using OCT angiography and a deep learning model. *Ophthalmol. Retina*. **9**, 378–385 (2025). 39461425 10.1016/j.oret.2024.10.017 PMC11972158

[18] Heinke, A. et al. Cross-instrument optical coherence tomography-angiography (OCTA)-based prediction of age-related macular degeneration (AMD) disease activity using artificial intelligence. *Sci. Rep.* **14**, (2024). 10.1038/s41598-024-78327-0 PMC11544254 39511248

[19] Morsy, M. S. et al. Effect of faricimab on optical coherence tomography angiography and artificial intelligence-based analysis in resistant choroidal neovascularization. *Ophthalmologica*. 1–10. 10.1159/000548690 (2025). 10.1159/000548690 41134744

[20] Fırat, M. et al. AI-based response classification after anti-VEGF loading in neovascular age-related macular degeneration. *Diagnostics*. **15**, 15 (2025). 10.3390/diagnostics15172253 PMC12428304 40941740

[21] Miere, A. et al. *Retina*. **39**, 548–557 (2019). 29210939 10.1097/IAE.0000000000001964

[22] Faatz, H. et al. The architecture of macular neovascularizations predicts treatment responses to anti-VEGF therapy in neovascular AMD. *Diagnostics (Basel)*. **12**, (2022). 10.3390/diagnostics12112807 PMC9688972 36428867

